# Cardiopulmonary bypass as a bridge for bronchial foreign body removal in a child with pulmonary artery sling: A case report

**DOI:** 10.1097/MD.0000000000026908

**Published:** 2021-08-13

**Authors:** Shuxian Li, Lei Wu, Meixia Huang, Junfen Zhou, Yingshuo Wang, Zhimin Chen

**Affiliations:** a Department of Pediatric Pulmonology, The Children's Hospital, Zhejiang University School of Medicine, National Clinical Research Center for Child Health, Hangzhou, Zhejiang, China; b Department of Endoscopy Center, The Children's Hospital, Zhejiang University School of Medicine, National Clinical Research Center for Child Health, Hangzhou, Zhejiang, China; c Department of Pediatrics, Wenling Maternal and Child Health Care Hospital, Wenling, Zhejiang, China.

**Keywords:** cardiopulmonary bypass, case report, flexible bronchoscopy, foreign body aspiration, pulmonary artery sling

## Abstract

**Rationale:**

Successful removal of an airway foreign body (FB) in some intractable cases can be very challenging, because of tracheal anomalies, unstable respiratory status of the patients, and the location of FB. The use of cardiopulmonary bypass (CPB) support for the treatment of a FB is extremely rare.

**Patient concerns:**

We present a case of a 39-month-old previously healthy girl who was admitted to our hospital for suspected FB aspiration (FBA). Initially, the attempt for removal of the FB by conventional bronchoscopy failed because of hypoxic intolerance.

**Diagnoses:**

Bronchoscopy revealed tracheal anomalies and subsequent computed tomography angiography demonstrated the presence of a pulmonary artery sling (PAS), which confirmed the diagnosis of PAS accompanied with FBA.

**Interventions:**

With the assistance of CPB, multidisciplinary treatment involving the respiratory, cardiothoracic and anesthetic teams were involved and the bronchial FB was removed by flexible bronchoscopy successfully and then PAS was corrected by surgical intervention.

**Outcomes:**

The patient remained asymptomatic, without shortness of breath or wheezing during the 15 months follow-up.

**Lessons:**

This case highlights that in a complicated case of FBA, bronchoscopy and computed tomography imaging are of great importance to achieve an accurate diagnosis, and a multidisciplinary treatment approach is essential for a satisfactory outcome. If the patient is unstable for bronchoscopy, CPB can be temporarily used in the stabilization of the patient to allow safe removal of the FB.

## 1 Introduction

Foreign body aspiration (FBA) is a common cause of respiratory problems in childhood, especially in children under 3 years.^[1]^ It causes significant morbidity and even mortality, and is responsible for accidental death in children under 1 year.^[1]^ Generally, foreign body (FB) can be extracted by bronchoscopic treatment.^[2]^ Rarely, some intractable cases may require the application of tracheotomy or the implementation of special life support, such as cardiopulmonary bypass (CPB), to facilitate FB extraction.^[3]^ Over half a century, CPB has been developed as an adjunctive or rescue therapy for multiple cardiac and various non-cardiac indications (e.g., respiratory or cardiac failure, cardiac or lung support, cardiopulmonary resuscitation, heart or lung surgery/transplantation).^[4]^ It provides adequate gaseous exchange by improving tissue oxygenation or carbon dioxide removal or temporary cardiorespiratory support to enable surgeons to perform various complex cardiac or challenging operations.^[5]^ Tracheal anomalies, which can remain asymptomatic and are incidental findings on imaging, are relatively uncommon.^[6]^ They may present with concurrent anomalies (e.g., vascular rings and slings) and the diagnosis is frequently delayed because of the rarity, making the treatment more complicated.^[7,8]^ Although the application of CPB has been rapidly expanded beyond traditional uses, the implementation of CPB in children with FBA, especially coexist with tracheal anomalies, to stabilize the child during endoscopic FB removal is scant. We experienced difficult FB removal in a child with PAS in which bronchoscopic retrieval under CPB support was eventually required.

## 2 Case presentation

**Figure 1:**
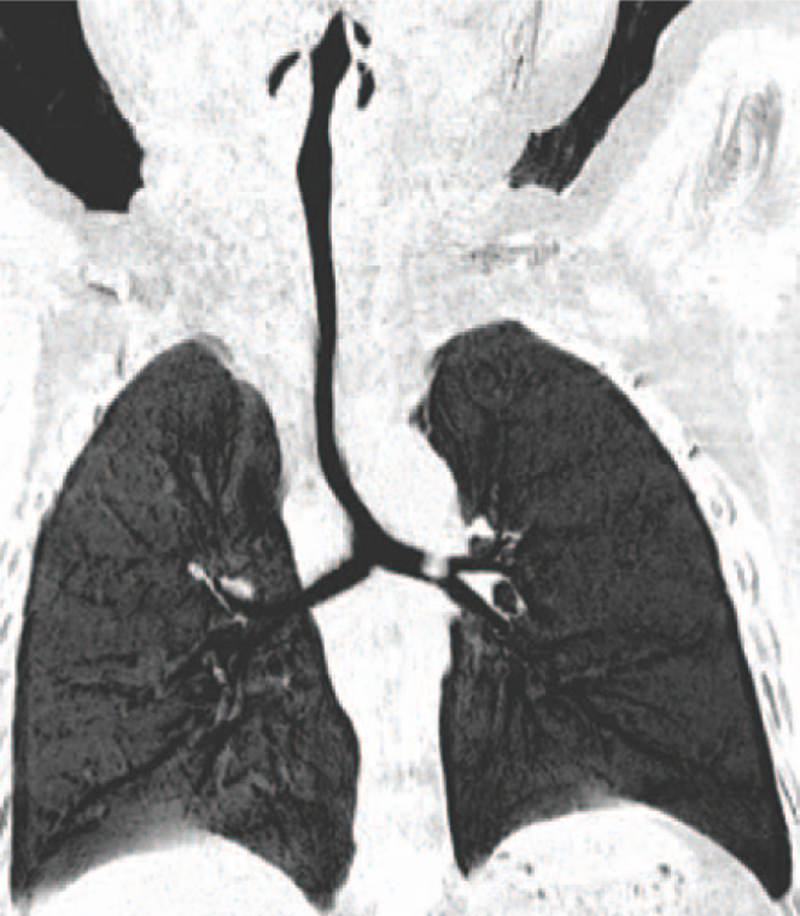
Three-dimensional CT image showing near total occlusion of the left main bronchus and left lung emphysema, a mild degree of tracheal stenosis, and abnormally low tracheal bifurcation at T6 level.

A 39-month-old girl, weighing 15.0 kg, was referred to our emergency department on August 18, 2019, with cough and wheezing for 2 days. Her parents recalled that the cough and wheezing started following a fit of chocking when the child was eating peanut. She was feeding well and gaining weight appropriately, and had unremarkable past medical history. Also, her family history was negative for any significant illnesses (e.g., asthma, tuberculosis). On admission, auscultation indicated diminished breath sounds as well as wheezing on the left lung. A subsequent chest three-dimensional (3D) reconstruction computed tomography (CT) scan showed nearly total occlusion of left main bronchus alongside left lung emphysema which strongly supported the suspicion of FBA, and a mild degree of tracheal stenosis, as well as abnormally low tracheal bifurcation at T6 level (Fig. 1).

**Figure 2:**
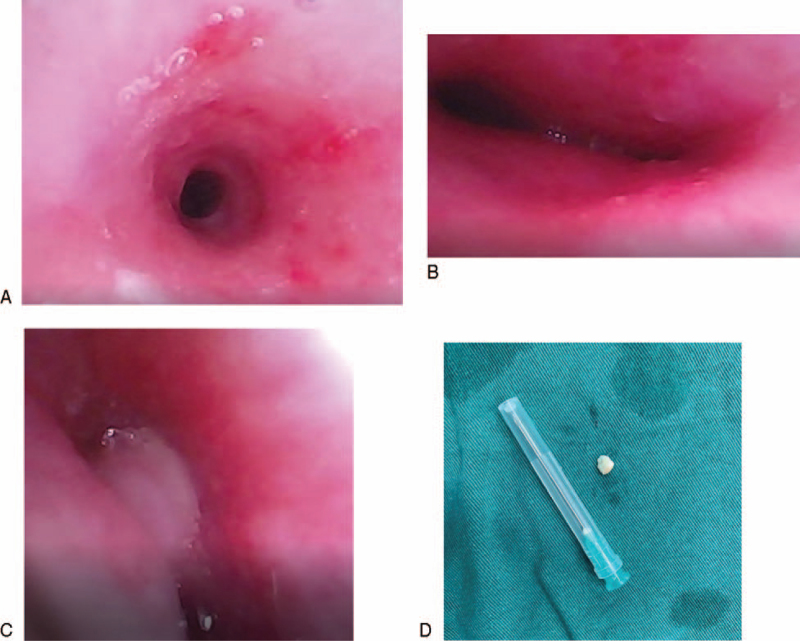
Bronchoscopy demonstrating airway dysplasia: (A) O-type tracheal cartilage; (B) the orifices of the main bronchi appeared to be smaller than normal size; (C) a white object embedded in the left main bronchus; (D) peanut fragment extracted by flexible bronchoscope with the help of cardiopulmonary bypass.

Immediately, the child underwent a bronchoscopic examination for definitive diagnostic and therapeutic purposes after preoperative preparation. Under general anesthesia, rigid bronchoscopy revealed subglottic stenosis, which made the passage of a 7.5 mm bronchoscope difficult. Flexible bronchoscopy using a 2.8 mm fiberoptic bronchoscope (FOB) was subsequently undertaken, and again the stenosis was encountered. The flexible bronchoscopy revealed a long stenotic segment of trachea that extended from approximately the entrance of the trachea to the bifurcation, especially in the middle and lower trachea, and O-shaped cartilaginous rings were visualized along the stenotic portion (Fig. 2A). The orifices of both mainstem bronchi appeared to be smaller than normal size (Fig. 2B). After suctioning off the endotracheal secretion, a white object was found embedded in the middle of left main bronchus (Fig. 2C). Unfortunately, extraction of the object had to be aborted, because of an inability to maintain adequate saturation during this procedure. Endotracheal secretion was sent for pathogen detection, including bacterial culture, mycobacterium tuberculosis, viral antigen test (e.g., adenovirus, respiratory syncytial virus, influenza A, influenza B, parainfluenza virus I, parainfluenza virus II, parainfluenza virus III). However, no pathogen was detected. Simultaneously, she was transported to the pediatric intensive care unit and administrated intravenous ceftriaxone and methylprednisolone, inhaled budesonide, ipratropium bromide, and salbutamol. However, the patient was unresponsive to these combined treatment and showed little improvement of the symptoms.

**Figure 3:**
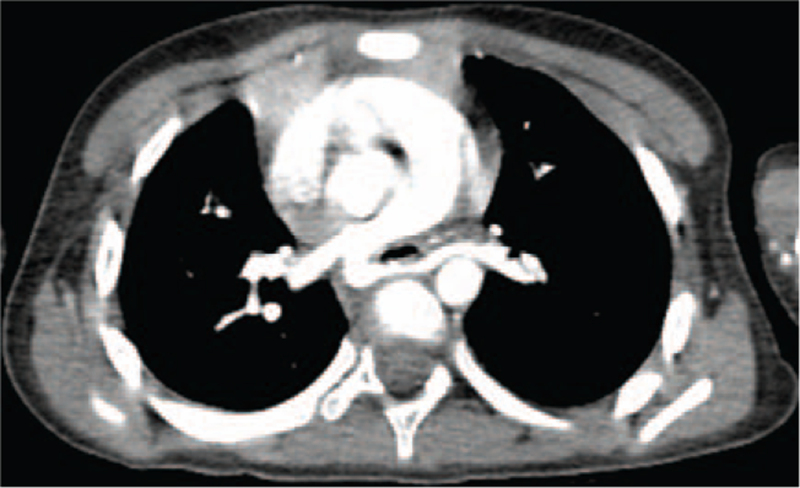
CT angiography depicting the origin of the left pulmonary artery arising from the posterior aspect of the right pulmonary artery and travelling between the esophagus and trachea towards supplying the left lung.

This situation motivated us to have a full reassessment to discriminate inflammatory, potential congenital, or vascular compression-related etiologies of the airway stenosis. For enlisting more clinical clues for differential diagnosis, we took the child's past history again. At this time, the parents recalled the child had experienced recurrent wheeze since the age of 3 months, but they presumed her acute onset of wheezing was from an upper respiratory infection and /or asthma. Then a cardiac CT angiography (CTA) was performed that demonstrated pulmonary artery sling (PAS). In detail, the origin of the left pulmonary artery (LPA) arose from the posterior aspect of the right pulmonary artery (RPA) and traversed between the trachea and esophagus towards the left lung, leading to the formation of a vascular ring and external tracheal compression (Fig. 3). These findings led to a new multidisciplinary surgical plan, that is, CPB during both FB removal by FOB and PAS repair. Firstly, bronchoscopy was performed on CPB, during which a peanut debris was successfully extracted from the left main bronchus (Fig. 2D). Then, the patient received the LPA reimplantation under CPB. In detail, the LPA was dissected from the RPA, mobilized, brought anteriorly, and reimplanted into the main pulmonary artery. The patient was weaned from CPB immediately after the surgery. The CPB lasted for 80 min. As cardiac deformity correction was satisfactory, she was weaned off of mechanical ventilation and transferred to the general ward by the 2^nd^ postoperative day. Consequently, she was discharged home on the 9^th^ postoperative day. The patient remained asymptomatic, without shortness of breath or wheezing on exercise during the 15 months follow-up.

## 3 Discussion

Although cases of FBA and its treatment have been widely described, case of PAS presenting with a bronchial FB is rarely reported. As discovered incidentally in our case, PAS can remain undetectable as they usually present with atypical symptoms (e.g., recurrent wheeze) mimicking those of common diseases, such as bronchial asthma, viral respiratory infection and FBA. Interestingly, our study showed PAS posed major obstacles during FB removal. The coexistence of stenotic airway segments, PAS, and FB lodged in the stenotic bronchus, further complicated the management of our case. Despite this dilemma, the interdisciplinary team involvement, comprehensive diagnostic evaluation helped in successful management of our case without any complications.

PAS is frequently accompanied by varying degrees of tracheal stenosis, which is caused by external tracheal compression by the arterial sling, or malacia of the tracheal rings or complete cartilaginous rings.^[9–11]^ Wells et al classified PAS into 2 main types and 2 subtypes, according to the thoracic level of the main carina (type I T4-5, Type II T5-6) and the presence (subtype A) or absence (subtype B) of an eparterial right upper lobe bronchus, respectively.^[12]^ Undoubtedly, our case was classified into PAS type IIB. The important investigation in our case included chest CT scan and bronchoscopy to identify the type, localization, severity of lesion, extent of tracheal stenosis and complete tracheal rings. In detail, bronchoscopy illustrated the tracheal and bronchial stenosis, and CT with 3D reconstruction provided useful anatomic images of the airway, and CTA showed a full assessment of the relationship between surrounding vascular structures and adjacent trachea. Moreover, because of the nonspecific symptoms of PAS, lack of clinical experience may lead to misdiagnosis. Utilization of CTA to rule out PAS should be considered as part of differential diagnosis of patients with trachea stenosis.^[13]^ Thus, considering tracheal stenosis is frequently associated with other congenital anomalies,^[6]^ a multi-modality approach, including bronchoscopy, CT with 3D reconstruction and CTA, is prerequisite for achieving a comprehensive diagnostic evaluation and optimization prior to surgery in patients with airway anomalies.

Nowadays, surgical correction is essential in children with PAS, and the preferred surgical approach for PAS have evolved towards using CPB via a median sternotomy.^[14]^ Briefly, under CPB support, the LPA is divided from the anomalous origin on the RPA and then reimplanted to the main pulmonary artery.^[15]^ However, because of lack of sufficient evidences, there is still no consensus on the optimal strategy for dealing with the tracheal stenosis.^[16]^ The clinical symptoms vary from almost asymptomatic to near fatal, mainly associated with the considerable variation of airway stenosis. In consideration of satisfactory growth, minor symptoms and the possibility of relief of narrow trachea after LPA reimplantation, the mild trachea stenosis in our case was managed conservatively and showed encouraging outcomes, implying not all pediatric patients with trachea stenosis require tracheal reconstruction. Similarly, patients with minor symptoms and a 4 to 6 mm tracheal diameter were advised to be managed conservatively.^[17]^ A tracheal diameter under 3 mm was recommended for surgical treatment in the study conducted by Huang et al.^[18]^ Oshima et al suggested that for patients with PAS and tracheal stenosis presenting moderate or more severe clinical symptoms, both PA reimplantation and tracheoplasty should be performed.^[19]^ Moreover, Hong et al demonstrated that the diameter/length ratio might be a more suitable index for tracheal intervention.^[20]^ These evidences imply that the treatment strategies for tracheal stenosis in PAS patients depended on the severity of clinical symptoms, diameter and length of the stenosis.

Bronchial FBA is a common condition in young children, and majority of the FBs can be successfully extracted by rigid bronchoscope or FOB. In our case, due to the difficulty of retrieving the FB and subsequent frequent hypoxemia during FB removal under interventional bronchoscopy, CPB was required as a bridge to facilitate the safe removal of the FB, suggesting management of FBA should be selected according to each patient's individual circumstances. Similarly, Park et al described 3 pediatric patients who underwent extracorporeal membrane oxygenation, a modified form of CPB, assisted FB removal.^[21]^ Deng et al also reported successful removal of an aspirated elliptic plastic ball in a 6-year-old girl under CPB support.^[3]^ These cases demonstrate that CPB support should be considered for temporary pulmonary support to stabilize the patient for endoscopic FB removal. Although we do not feel that CPB availability is a prerequisite for FB removal (as the need is so rare), the use combined techniques (e.g., FOB under CPB) is required depending on the patient's status (e.g., hypoxic intolerance, instable hemodynamics, severe respiratory failure). With its inherent associated potential complications (e.g., hemorrhage due to anticoagulation, hemolysis, and embolization), the decision to use CPB cannot be taken lightly and must be a joint decision by the specialist teams involved. In our case, the child not only had FBA but also had complete tracheal ring and associated cardiovascular malformation, further complicating the management. These 3 factors determined multidisciplinary input and justified the use of CPB. In consideration of the desaturation from PAS-related airway stenosis and bronchospasm during FB removal, and the demand of CPB for PAS repair, the multidisciplinary consensus from otolaryngologist, pulmonologist, cardiovascular surgeon, thoracic surgeon, and anesthesiologist is required for endoscopic removal and PAS repair. Of note, the availability of a trained CPB team is essential for the expedient application of the technique. Ultimately, safe and timely removal of the FB was achieved based on the strong teamwork among these departments, implying multidisciplinary cooperation is essential for ensuring a good patient outcome.

Both congenital tracheal and vascular anomalies can cause various non-specific respiratory symptoms, including cough, wheezing, recurrent respiratory infections, stridor, noisy breathing, dyspnea, respiratory distress, and cyanosis. Still, some anomalies can be asymptomatic and found by accident just as our case. Tracheal anomalies not only are relatively rare in the general pediatric population, but are also often accompanied by congenital disorders, including vascular anomalies (e.g., PAS), congenital heart disease (e.g., ventricular septal defects), or gastrointestinal defects.^[22]^ In our case, O-shaped cartilaginous rings and PAS were not initially suspected because of the rarity of the lesion and the chief complaint of suspected FBA. For these concerns, an early and accurate diagnosis requires experience and a high index of clinical suspicion.^[23]^ Furthermore, PAS is often associated with congenital tracheal stenosis (e.g., complete tracheal cartilage ring), making the diagnosis and treatment more challenging.^[11]^ Collectively, these data alert pediatricians to realize that respiratory symptoms sometimes can be multifactorial and attributed to 2 more organ systems rather than the respiratory system alone.

## 4 Conclusions

Bronchoscopy is the essential diagnostic and therapeutic maneuver for FBA. For special cases with FBA, when the patients are unstable to undergo bronchoscopy, the utilization of the CPB or with CPB on standby prior to airway intervention facilitates surgical procedures. Findings indicative of tracheal anomalies require further diagnostic bronchoscopy and imaging studies (e.g., CTA) in the workup.

## Acknowledgments

We appreciate the support from the child enrolled in this study and her guardians. We obtained written informed consent from the patient's parents for the publication of any potentially identifiable images. Also, we gratefully acknowledge all the doctors and nurses who involved in the treatment.

## Author contributions

**Conceptualization:** Yingshuo Wang, Zhimin Chen.

**Funding acquisition:** Shuxian Li.

**Supervision:** Yingshuo Wang, Zhimin Chen.

**Writing – original draft:** Shuxian Li, Lei Wu.

**Writing – review & editing:** Shuxian Li, Lei Wu, Meixia Huang, Junfen Zhou, Yingshuo Wang, Zhimin Chen.
